# Transcriptomic analysis reveals the mechanism of the alleviation of salt stress by salicylic acid in pepper (*Capsicum annuum* L.)

**DOI:** 10.1007/s11033-022-08064-y

**Published:** 2022-11-23

**Authors:** Jing Ma, Ying Wang, Li-yue Wang, Duo Lin, Yanjie Yang

**Affiliations:** 1 Key Laboratory of Horticultural Plant Genetic Improvement and Breeding of Qingdao, College of Horticulture, Qingdao Agricultural University, 700 Changcheng Road, Qingdao, 266109 China; 2 College of Science and Technology, Henan University, Kaiyuan Avenue, Luoyang, 263471023 China

**Keywords:** Pepper, Salt tolerance, Salicylic acid, Alleviation, TFs

## Abstract

**Background:**

The growth and yield of pepper (*Capsicum annuum* L.) is often affected by the critical salt stress. Salicylic acid (SA) can improve plants’ stress tolerance by promoting growth and regulating ion absorption and transportation.

**Methods and results:**

To uncover the alleviated mechanism of salt stress by SA in pepper, we conducted morphological, physiological, cytological, and transcriptomic analyses under a single SA treatment and NaCl with and without SA pre-treatment for 9 days. Seedlings under NaCl treatment showed yellow shrunken leaves, this tatus were alleviated by NS treatment (NaCl with SA pre-treatment). Compared with plants under NaCl treatment, those in the NS treatment showed reduced lipid peroxidation, and significantly increased contents of chlorophyll and osmotic regulators (proline, soluble sugars). Treatment with SA balanced the Na^+^/K^+^ ratio. We conducted transcriptome sequencing and identified differentially expressed genes (DEGs) contributing to alleviation of salt stress by SA in pepper. Besides photosynthesis related genes, GO and KEGG analyses revealed that the DEGs were enriched in ‘sequence-specific DNA binding’, ‘transcription regulator activity’ and ‘DNA binding transcription factor activity’ by GO terms. And our results showed that TFs, such as MYB, bZIP, BBX, AP2/ERF, NAC, etc., probably make a great contribution in the alleviation of salt stress by SA.

**Conclusions:**

These results reveal that SA can improve plants’ stress tolerance by balancing ion absorption, gene expression and transcriptional regulation, which provide new ideas and resources for subsequent research on the mechanism of salt tolerance in pepper.

**Supplementary Information:**

The online version contains supplementary material available at 10.1007/s11033-022-08064-y.

## Introduction

Soil salinity is a major factor limiting plant productivity in many cultivated areas. More than 40% of irrigated agricultural land worldwide is predicted to be affected by salinity [1]. At present, salinization of soil is becoming increasingly serious as a result of global climate change, industrial pollution, the development of irrigated agriculture, and the improper use of chemical fertilizer. Thus, soil salinization threatens the sustainable development of agriculture. Salinity is an important environmental factor affecting plant growth and yield, and high salt levels can reduce plant growth or even cause death. Salinity stress leads to ion imbalances and toxicity because plants absorb excess toxic ions from the soil [2]. Salt stress affects plant growth in various ways, but drought and salt-sensitive plants are seriously damaged by the disruption of the balance between potassium and sodium ions (K^+^ and Na^+^) in cells, which affects many physiological processes [3]. Maintenance of a low Na^+^/K^+^ in plant cells is considered to be a key salt-tolerance trait [4, 5]. Some plants have evolved multiple adaptive strategies to maintain cellular and whole-plant Na^+^/K^+^ homeostasis to adapt to salt stress [6]. Thus, controlling the ion concentration in cells is important for homeostasis. In plants, under salt stress, Na^+^ can enter the cells via several pathways, and it can become toxic to cytosolic enzymes at high concentrations. To keep the cytosolic Na^+^ at low level and maintain osmotic balance, plants employ different strategies to prevent and adapt to high Na^+^ concentrations: active Na^+^ efflux, Na^+^ compartmentalization in vacuoles, and prevention of Na^+^ influx [7]. A high Na^+^ concentration in plant tissues can interfere with the function of K^+^. In plants, K^+^ participates in a series of metabolically related enzymatic reactions, and a high external Na^+^ concentration competes for K^+^ and disrupts cellular homeostasis [8]. Plant membrane transporters regulate Na^+^ uptake and transport to control intracellular ion homeostasis in halophytes under high-salt conditions [8]. The Na^+^(K^+^)/H^+^ antiporters in plant cells are an important group of proteins that remove Na^+^ from the cytoplasm to maintain a low cytoplasmic Na^+^ concentration.

Salicylic acid (SA) is an endogenous growth regulator [9] and belongs to a group of phenol compounds. It participates in the regulation of physiological processes [10] and prevents damage to plants caused by pathogens, as well as salt stress [11]. SA can promote the growth rate of plants under salt stress, modulate leaf gas exchange with the atmosphere and plant carbohydrate metabolism to alleviate salt stress [12–15], also by regulating the absorption and transport of ions and stabilize the ion balance in cells [16]. In addition to its important role in physiological processes, including enhancing nitrogen metabolism, inducing proline and glycinebetaine, and triggering enzymes of defence compounds synthesis [17, 18]. The application of exogenous SA also can improve photosynthetic rate under salt stress, resulting from the capacity to increased rubisco activity and K^+^ absorption [19, 20], causing to ATP content raising and optimum K^+^/Na^+^ ratio maintaining in plants [21]. Thus, SA may be involved in the alleviation of salt stress in plants.

Pepper is one of the three most important Solanaceous vegetable crops worldwide, and it is generally considered to be salt sensitive [22]. Peppers are often affected by abiotic stresses such as drought and salinity during growth, and salinity severely affects the yields and quality of pepper in dry and semi-arid areas [23]. Pepper is regarded as sensitive to moderately sensitive to salt [24, 25]. Pepper plants exposed to salt show decreased growth and disruptions in membrane permeability and ion balance [26]. Transcriptome analysis is a useful tool for understanding the molecular mechanism of the alleviation of salt stress by SA through enrichment of DEGs. Moreover, transcription factors (TFs) play essential roles in salt stress tolerance, transcriptome analysis can achieve key TFs accurately and efficiently.

SA is important for plant growth and enhance stress tolerance, however, the expression of genes of the SA-mediated alleviation of salt stress, and how SA coordinate pepper salt stress acclimation at molecular levels is largely undisclosed. In this study, we treated pepper seedlings with NaCl, SA, or NS, or Hoagland’s nutrient solution (CK) for 9 days, then the morphological, physiological, and cytological analyses were investigated to compare the difference of the untreated or treated samples. We also performed RNA seq analyses on the leaves of these seedlings at 3 days under various treatments, the DEGs were thoroughly analysed to further explore the molecular mechanism of the alleviation of salt stress by SA.

## Materials and methods

### Plant material and growth conditions

Healthy and viable seeds of pepper (*C. annuum* L.) were surface sterilized using sodium hypochlorite (10%, v/v) for 15 min, then washed with distilled water. The seeds were germinated in a Petri dish on filter paper, and the treatment liquid was changed every day. Healthy germinated seedlings were transferred into a seedling tray filled with peat, vermiculite, and perlite (vol/vol 1:1:0.5). Seedlings were grown in a growth chamber at 25/16 °C (16-h light/8-h dark photoperiod) with relative humidity of 70%. After 50 to 60 days, a hydroponic system was established using pepper seedlings with uniform growth. Four treatments were applied: CK (Hoagland’s nutrient solution), NaCl (100 mM), SA (0.15 mM), and NaCl + SA (NS) (SA: 0.15 mM + NaCl: 100 mM). The total treatment period was 9 days. Samples were taken for analysis after treated 3, 6, and 9 days. At each sampling time, samples from three replicates, three seedlings of each replicate were analysed simultaneously. A portion of each sample was frozen in liquid nitrogen and then stored frozen until further analyses.

### Microscopy analysis of leaf structure

Leaf pieces were examined under a light microscope (Nikon Eclipse E100, Tokyo, Japan) to characterize stomatal morphology and density and to observe the arrangement of cells within the leaf. Samples were prepared for microscopy according to the method with minor modifications [27] and at least five independent biological replicates were examined.

### Physiological characterization and determination of ion contents in pepper under salt stress

Chlorophylls were extracted from leaf discs in 75% alcohol and stored in the dark for 24 h, the supernatant was measured at 646 nm and 663 nm, and the chlorophyll content was calculated according to Lichtenthaler [28]. The level of lipid peroxidation was determined by measuring the malondialdehyde (MDA) concentration using the thiobarbituric acid method [29]. Proline (Pro) content was determined using the method described by Bates et al. [30]. Soluble sugars were quantified as described elsewhere [31]. The Na^+^ and K^+^ contents in leaves were determined as described elsewhere [32]

### RNA isolation and cDNA library construction

Total RNAs were isolated from pepper leaves harvested on day 3 of the various treatments using an RNA Prep Pure Plant kit (Tiangen Biotech., Beijing, China). The 12 RNA samples (C1, C2, C3, N1, N2, N3, S1, S2, S3, NS1, NS2, and NS3) were sent to Novogene-Bioinformatics Technology Co. Ltd. (Tianjin, China) for library construction and sequencing.

The integrity of RNA was confirmed using an RNA Nano 6000 Assay kit and the Bioanalyzer 2100 system (Agilent Technologies, Palo Alto, CA, USA). Sequencing libraries were generated using the NEBNext® UltraTM RNA Library Prep Kit for Illumina® (NEB, Beverly, MA, USA). Briefly, mRNA was purified from total RNA using polyA oligo-attached magnetic beads and fragmented using Illumina proprietary fragmentation buffer at 94 ℃ for 15 min. First-strand cDNA was synthesized using random hexamer primer and M-MLV Reverse Transcriptase (RNase-H). Second-strand cDNA was then synthesized. These cDNA fragments were then subjected to an end-repair process. The insert size of the library was restricted to approximately 250 ~ 300 bp using PCR products purified with the AMPure XP system (Beckman Coulter, Beverly, MA, USA).

### Quality control and sequence assembly

Raw data (raw reads) in fastq format were first processed using in-house perl scripts. Clean data (clean reads) were obtained by removing reads containing adapters, reads containing poly-N, and low-quality reads from the raw data. We then calculated the Q20, Q30, GC-content, and sequence duplication levels for the clean data. All the downstream analyses were based on high-quality clean data. The retained high-quality reads were mapped to the pepper reference genome *C. annuum*. L_Zunla-1_v2.0. Hisat2 v2.0.5 was used to build the index for the reference genome and to align paired-end clean reads to the reference genome. Expression level of each gene was calculated by using reads per kb per million reads (RPKM) method [33].

### Determination of differentially expressed genes (DEGs)

To quantify the abundance of gene transcripts, all reads from samples were mapped onto the reference transcriptome by feature Counts (1.5.0-p3) [34]. We used the FPKM value of each gene, which was calculated based on the length of the gene and the number of reads mapped to that gene. Differential expression analysis between two groups was performed based on DESeq method by using the DESeq2 R package (1.16.1). The resulting P-values were adjusted using the Benjamini and Hochberg’s approach for controlling false discovery rates. Finally, the DEGs were screened based on |log2(FoldChange)|> 1 and padj < 0.05 as a threshold.

### *Gene ontology *(GO)*enrichment and kyoto encyclopedia of genes and genomes pathway analyses*

The GO enrichment analysis of DEGs was implemented using TBtools v1.06 [35], where a corrected *P*-value < 0.05 was indicative of a significantly enriched GO term. In the KEGG enrichment analysis, the cellular metabolic and biochemical pathways and potential biological behaviour of differentially expressed genes (DEGs) were examined. We used clusterProfiler R package to test the statistical enrichment of DEGs in KEGG pathways. A corrected *P*-value < 0.05 was set as the threshold to determine significant enrichment of gene sets.

## Results

### The growth of pepper seedlings alteration in response to salt stress and salicylic acid

**Fig. 1:**
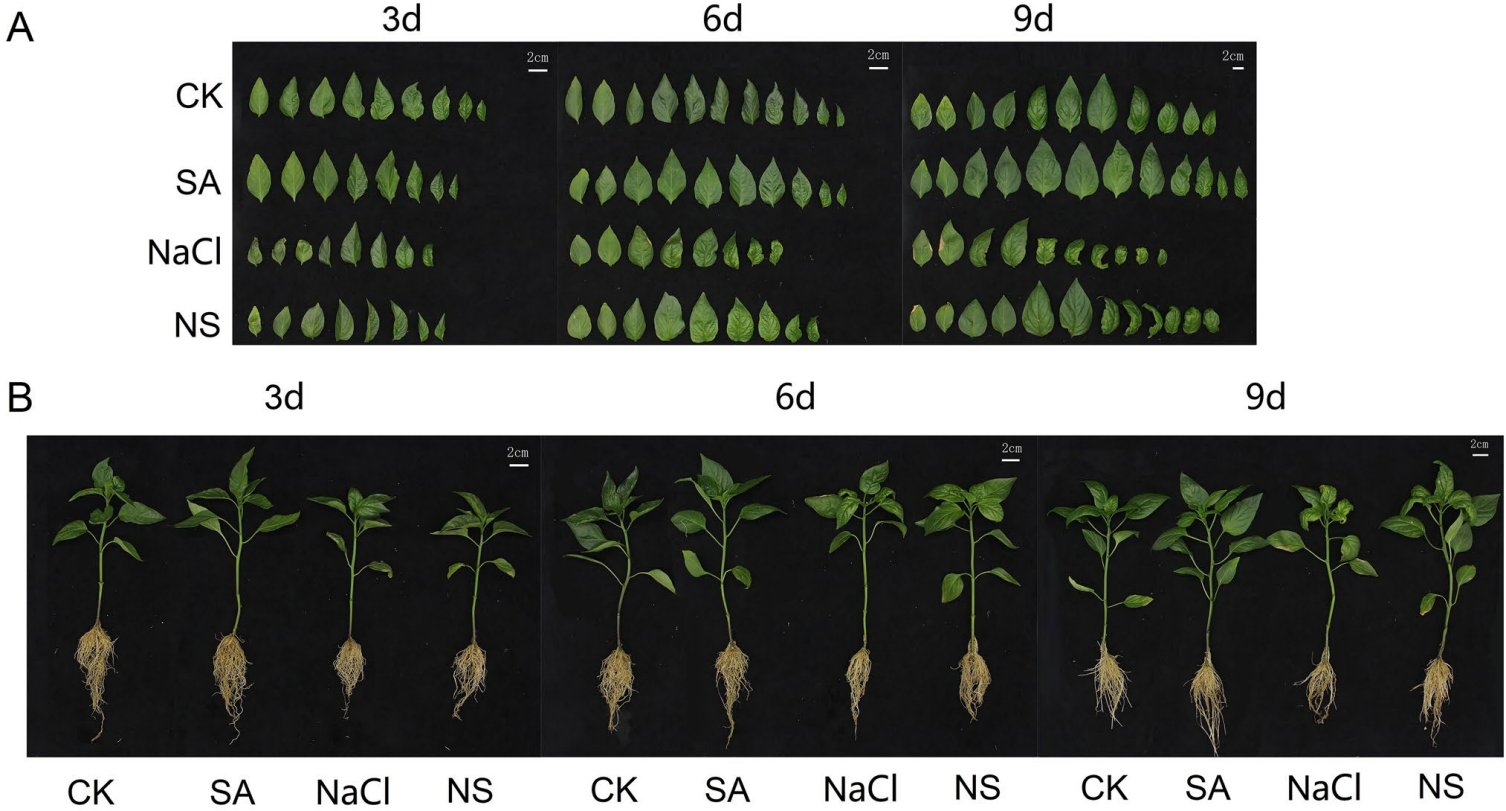
Pepper plants grown in nutrient solution with 100 mM NaCl and/or 0.15 mM SA for 3, 6, and 9 days. **A** Overall external characteristics of pepper plants in different treatments. **B** External morphology of pepper leaves from different treatments. CK: Hoagland’s nutrient solution; NaCl: 100 mM NaCl; SA: 0.15 mM SA; NS: 100 mM NaCl + 0.15 mM SA

After 3-, 6-, and 9-day treatments, seedlings treated with SA and those in control showed vigorous growth. However, those plants treated with NaCl grown weakly resulting in curly and yellow leaves, while the seedlings in the NS treatment showed sturdier phenotypes similar to that of control (Fig. 1A). This result indicated that exogenous SA alleviated NaCl stress in pepper seedlings to some extent. As shown in Fig. 1A, the leaves of seedlings treated with SA had a darker green colour than that of control, while the leaves of seedlings treated with NaCl were yellow and shrunken, and these symptoms worsened during the treatment time. In the NS treatment, the leaf colour and size were comparable to those of seedlings in the CK (Fig. 1B).

**Fig. 2:**
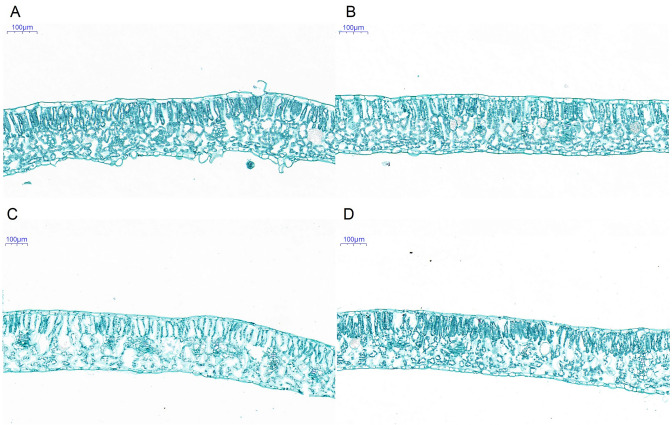
Ultrastructure of leaves of pepper plants grown for 3 days in nutrient solution with 100 mM NaCl and/or 0.15 mM SA. **A** SA, **B** CK, **C** NaCl, **D** NS. CK: Hoagland’s nutrient solution; NaCl: 100 mM NaCl; SA: 0.15 mM SA; NS: 100 mM NaCl + 0.15 mM SA

To further explore these morphological differences among treatments, we observed the ultrastructure of the young leaves. The leaf cells were tightly packed and the upper and lower epidermal cells were arranged regularly under SA treatment (Fig. 2A). In contrast to none treatment, the cell density in leaves was slightly lower under SA treatment (Fig. 2B). However, compared with seedlings of non-treatment and under SA treatment, those under salt treatment had more irregular cell row junctions and larger gaps within the leaves (Fig. 2C). Compared with the NaCl treatment, the NS treatment resulted in a tighter and more orderly arrangement of leaf cells, and smaller intercellular spaces. This result provided further evidence that SA can alleviate the damage caused by salt stress to leaves (Fig. 2D).

### Biochemical parameters in pepper leaves under different treatments

**Fig. 3:**
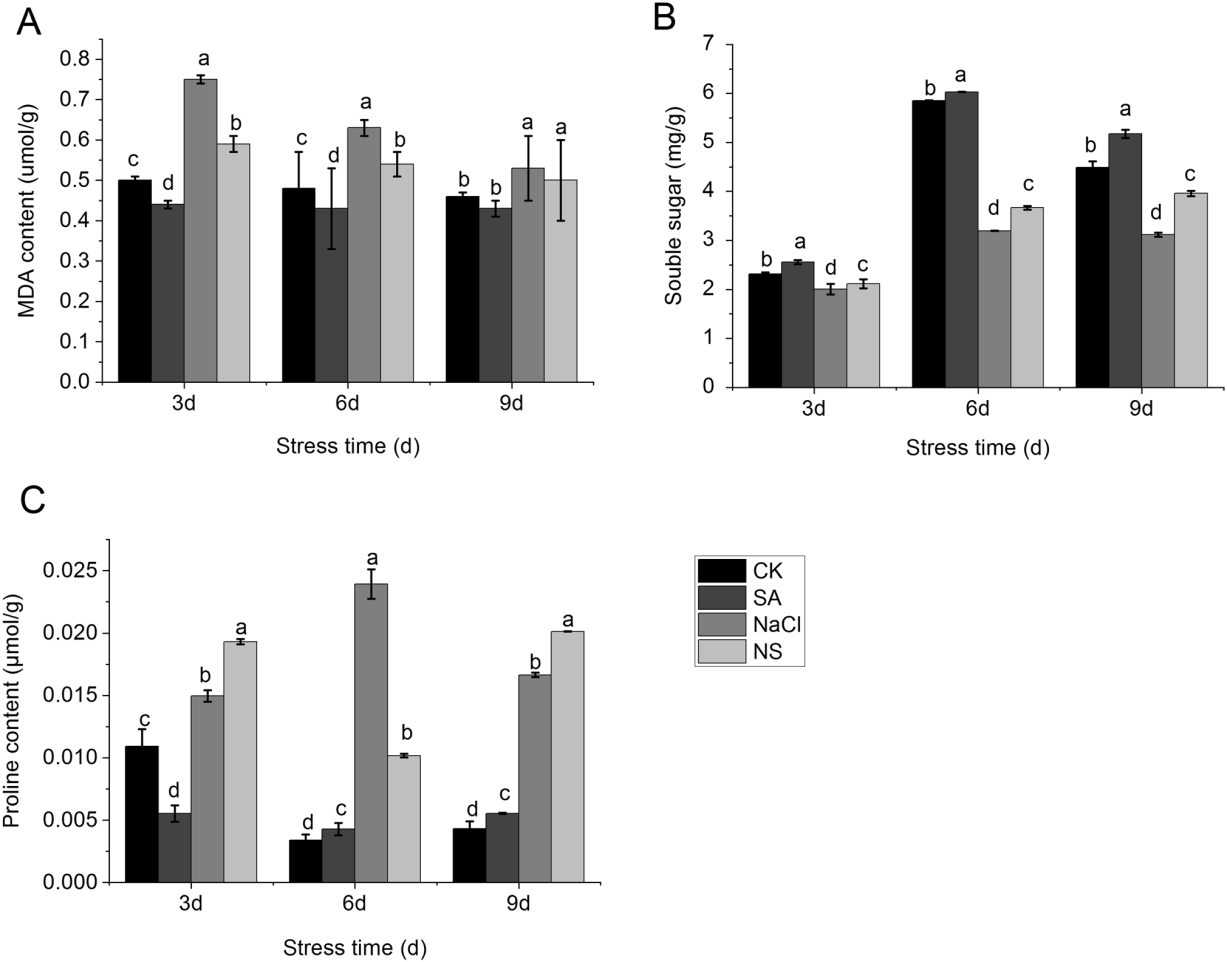
Effect of various treatments on MDA content (**A**), soluble sugars content (**B**), and proline content (**C**) of pepper plants grown for 3, 6, and 9 days in nutrient solution with 100 mM NaCl and/or 0.15 mM SA. Data are means of three replicates with SE. Different letters indicate significant differences between four different treatments at same stage at P < 0.05. CK: Hoagland’s nutrient solution; NaCl: 100 mM NaCl; SA: 0.15 mM SA; NS: 100 mM NaCl + 0.15 mM SA

To determine the physiological responses of pepper seedlings to salt and NS treatment, the proline contents, MDA contents, chlorophyll contents and soluble sugar contents were detected. With 3-, 6- and 9-day treatments, the proline contents of pepper leaves were much higher under the NaCl treatment and the NS treatment than that without any treatment. Treatment with SA alone resulted in a higher proline content than that of CK at day 6 and 9 but not at day 3 (Fig. 3A). The extent of lipid peroxidation, as determined by measuring MDA content, which was significantly induced under salt treatment, and was inhibited by NS treatment (Fig. 3B). The soluble sugar in the leaves were induced by SA treatment, but dramatically decreased under salt stress treatment, then enhanced by NS treatment (Fig. 3C).

**Fig. 4:**
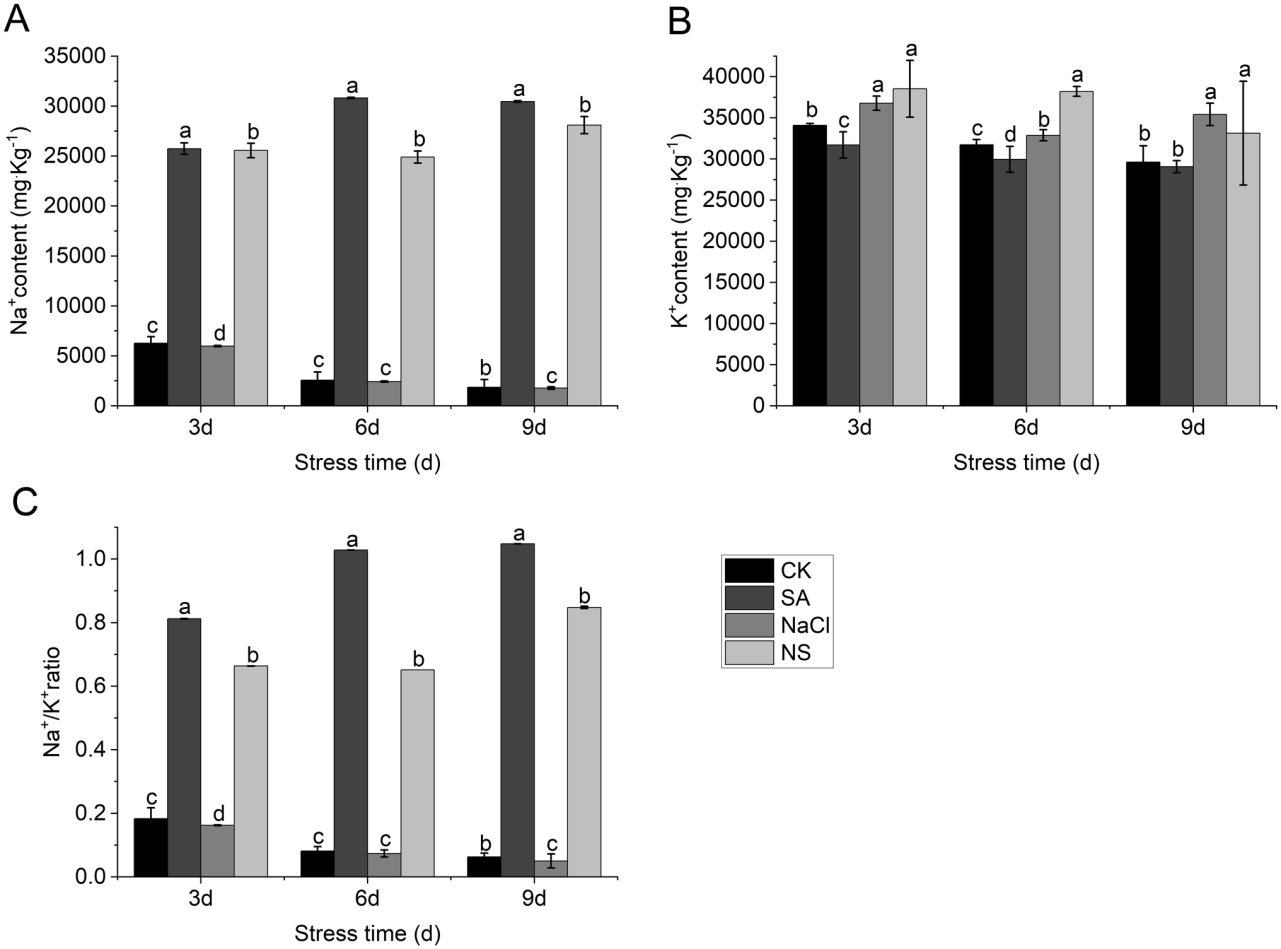
Effect of SA on ion contents and Na^+^/K^+^ ratio in pepper seedlings under NaCl stress. Na^+^ content (**A**) and K^+^ content (**B**) and Na^+^/K^+^ ratio (**C**) in leaves of pepper seedlings grown for 3, 6, and 9 days in nutrient solution with 100 mM NaCl and/or 0.15 mM SA. Data are means of three replicates with SE. Different letters indicate significant differences between four different treatments at same stage at P < 0.05. CK: Hoagland’s nutrient solution; NaCl: 100 mM NaCl; SA: 0.15 mM SA; NS: 100 mM NaCl + 0.15 mM SA

In addition, we also investigated the Na^+^ and K^+^ contents to detect the ion absorption under salt with and without SA treatment, the K^+^ content was higher than the Na^+^ content in all pepper seedlings under all treatment. Compared with CK, the NaCl treatment resulted in slightly lower K^+^ contents in pepper seedlings, and the SA and NS treatments resulted in slightly higher K^+^ contents (Fig. 4A). Compared with CK, the NaCl treatment resulted in greatly increased Na^+^ contents in pepper seedlings, but this was alleviated to some extent in the NS treatment. For example, the Na^+^ content in pepper seedlings was 30,814.1 mg/kg on day 6 of the NaCl treatment, but 23.8% lower in the NS treatment (Fig. 4B). Because the Na^+^ contents in seedlings were much higher in the NaCl and NS treatments than in CK and the SA treatment, the Na^+^/K^+^ differed markedly between these two groups. The Na^+^/K^+^ in the NaCl treatment was 0.81, 1.03, and 1.05 on day 3, 6, and 9, respectively, compared with 0.66, 0.65, and 0.85, respectively, in the NS treatment and < 0.2 at all sampling times in CK and the SA treatment (Fig. 4C). Thus, the NS treatment led to a significantly lower Na^+^/K^+^ than that in the NaCl treatment. This showed that SA could alleviate the damage caused by salt stress, and maintain the balance of Na^+^ and K^+^.

### Illumina sequencing and assembly

The numbers of raw reads generated from samples in the four treatments were 5.63, 6.12, 6.48, and 6.17 million, respectively. The average proportions of Q20, Q30, and GC across the 12 samples were 97.82%, 93.56%, and 42.70%, respectively (Table S1). We obtained approximately 73.24 million total reads, of which approximately 72.39 million passed the Illumina quality filtering threshold, yielding a quality rate of > 98.84%. This result indicated that the throughput sequencing data were sufficiently accurate to allow for further analyses.

Reference-based transcriptome assemblies of RNA-seq data were performed using the reference pepper genome sequence. Mapping results showed that there were about 52,889,856 (95.13%), 58,029,558 (95.83%), 61,484,630 (95.97%), and 58,272,960 (95.36%) unigenes in the CK, NaCl, SA, and NS libraries, respectively (Table S2), corresponding to 90.21%, 91.21%, 90.72%, and 89.84% of reads mapped to the reference genome, respectively. These reads were then used for reference-guided assembly and differential expression analysis.

### Identification of DEGs involving in the alleviation of salt stress by SA in pepper

**Fig. 5:**
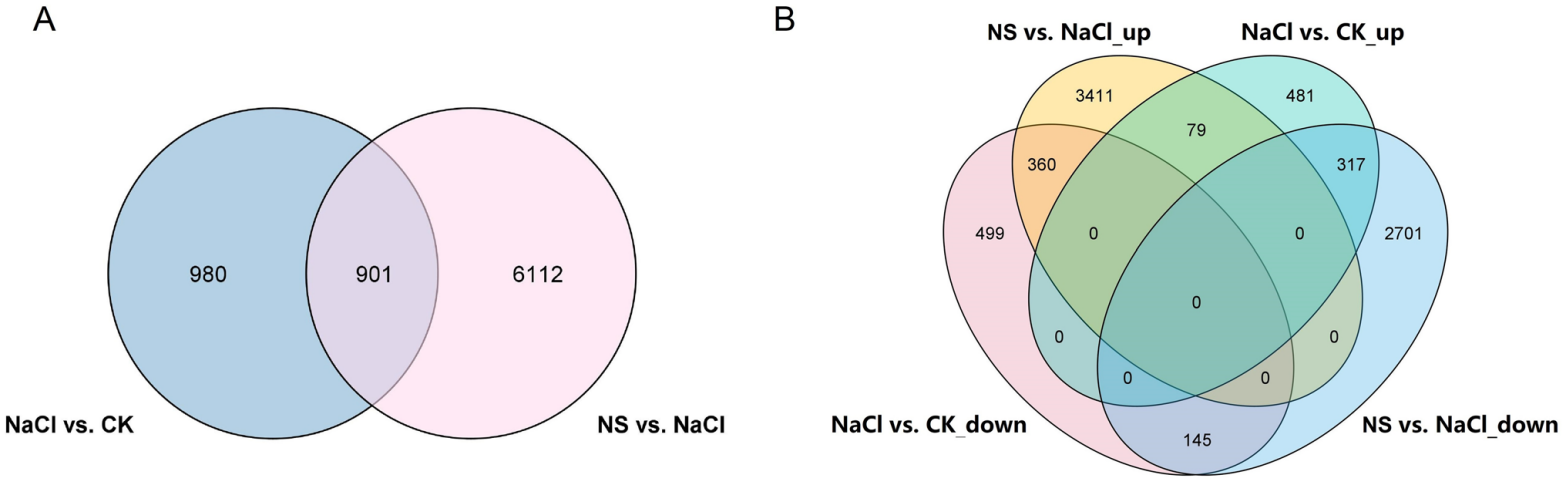
Venn diagram of differentially expressed genes (DEGs). Numbers of each circle show the number of stress responsive DEGs that are uniquely (inside of non-overlapping part) or commonly (inside of overlapping part) regulated. Diagram **A** shows the total DEGs involved in the alleviation of salt stress by SA in pepper were 901, **B** shows the different type of DEGs involved in the alleviation of salt stress by SA in pepper

To understand the mechanism of the alleviation of salt stress by SA in pepper, 901 related DEGs were selected by the intersection of NaCl vs. Ck DEGs and NS vs. NaCl DEGs (Fig. 5A). In addition, to distinguish the different function of the 901 selected DEGs, we also detected the intersection of NaCl vs. CK_up and NS vs. NaCl _down, NaCl vs. CK_down and NS vs. NaCl _up, NaCl vs. CK_up and NS vs. NaCl _up, NaCl vs. CK_down and NS vs. NaCl _down (Fig. 5B). And there were 317, 360, 79 and 145 identified in these intersections, respectively (Fig. 5B).

### Go enrichment analysis of identified DEGs

**Fig. 6:**
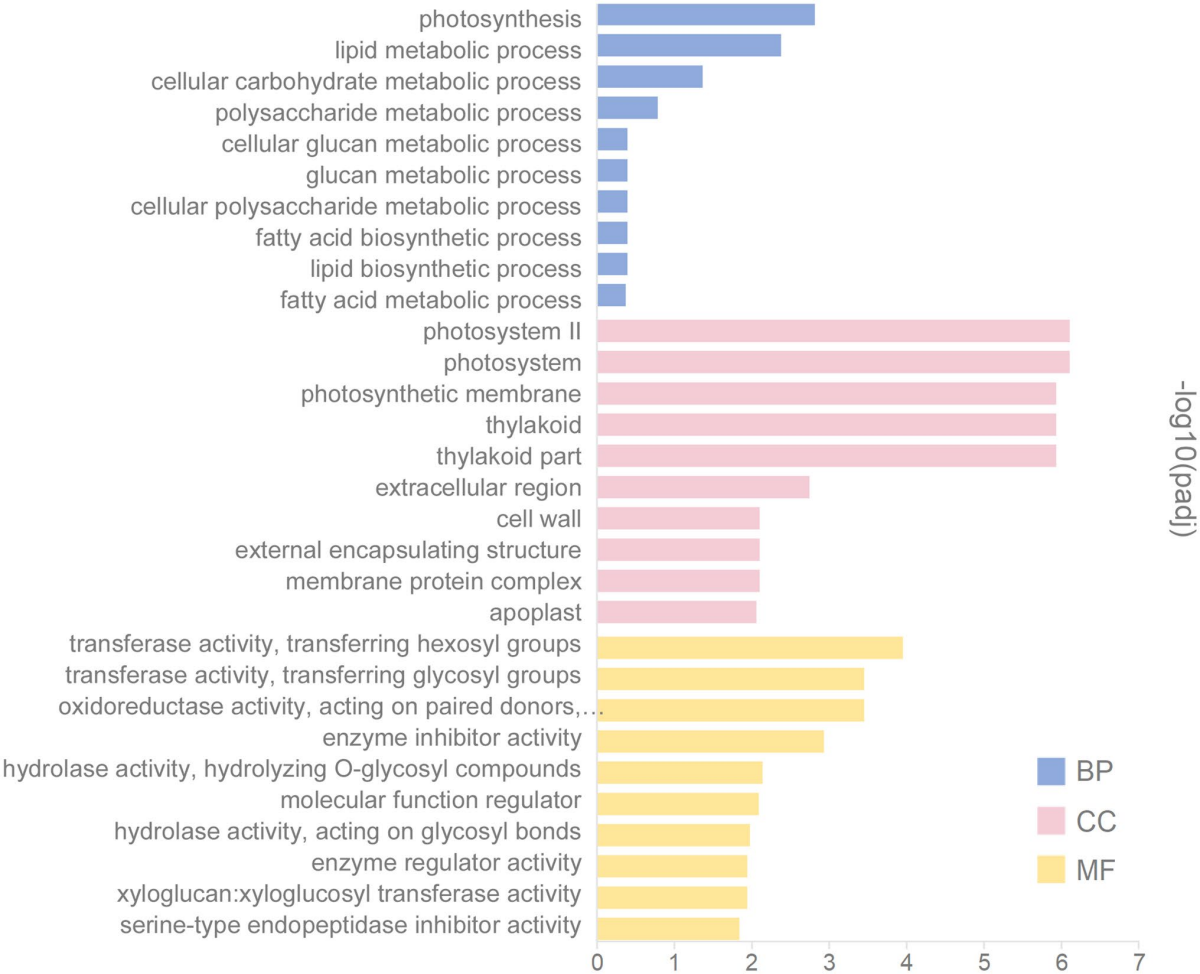
Functional classification of GO terms into three categories: *MF* molecular function, *BP* biological process, and *CC* cellular component. X-axis indicates GO term. Y-axis represents the level of significance of GO term enrichment

We performed a GO enrichment analysis to better understand the potential functions of DEGs involved in the alleviation of salt stress by SA in pepper. According to the results of sequence alignments, in total, there are 35 significant GO terms distributed in each three GO categories, namely molecular function (3 members), biological processes (12 members), and cellular components (20 members) (Fig. 6 and Table S3). And the top 10 GO terms of each three GO categories were identified in Fig. 6.

The most abundant GO terms were ‘photosystem II’ and ‘photosystem’, which belong to ‘cellular components’ category. And in ‘cellular components’ category another three abundant GO terms were ‘photosynthetic membrane’, ‘thylakoid’ and ‘thylakoid part’. In ‘biological processes’ category, most DEGs concentrated in the three GO terms: ‘photosynthesis’, ‘lipid metabolic process’ and ‘cellular carbohydrate’ metabolic process. In the ‘molecular function’ category, the largest number of DEGs involved in ‘transferase activity, transferring hexosyl groups’, ‘transferase activity, transferring glycosyl groups’, ‘oxidoreductase activity, acting on paired donors, with oxidation of a pair of donors resulting in the reduction of molecular oxygen to two molecules of water’ and ‘enzyme inhibitor activity’. These results indicated that most of the identified DEGs were related to fundamental processes associated with photosynthesis.

### KEGG enrichment analysis of identified DEGs

**Fig. 7:**
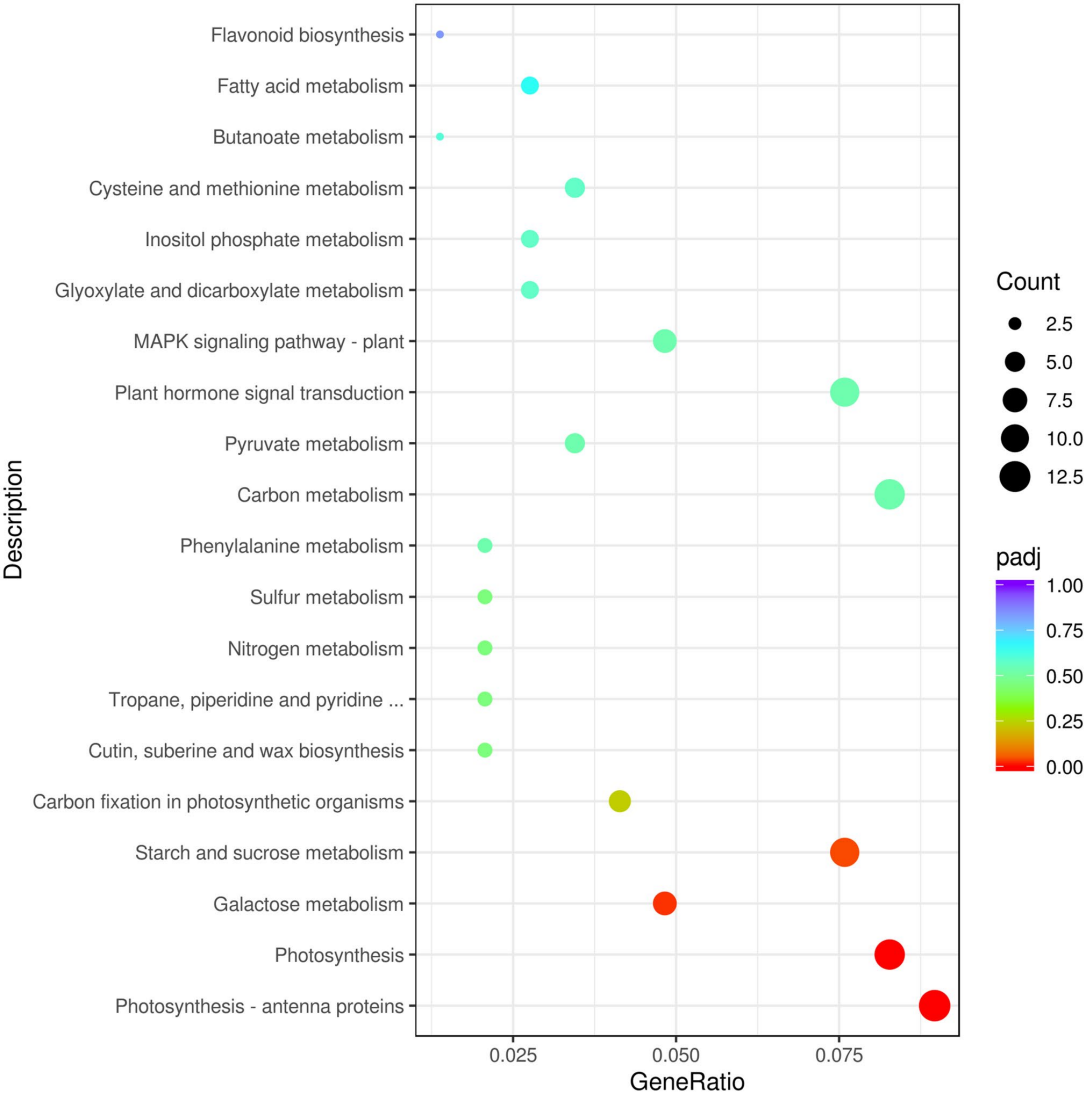
KEGG enrichment of annotated DEGs across three comparisons. Y-axis indicates KEGG pathway, X-axis indicates enrichment factor. High q values are shown in blue, low q values are shown in red

KEGG pathway enrichment analysis was performed to categorize the biological functions of identified DEGs. 145 of 901 identified DEGs were allocated to 66 KEGG pathways (Table S4). Among the significantly enriched pathways were ‘Photosynthesis—antenna proteins’, ‘Photosynthesis’, ‘Galactose metabolism’ and ‘Starch and sucrose metabolism’ (Fig. 7). Therefore, these DEGs directly or indirectly involved in photosynthesis or encoding proteins that are likely related to the function of SA to alleviate salt stress. With GO annotation together, these results indicated that gene involved in photosynthesis may play an important role in the alleviation salt stress by SA in pepper.

### Global analysis of TF families in pepper of alleviation of salt stress by salicylic acid

As is known, TFs play an important part in tolerance of salt stress. We performed a global TF classification of the 901 DEGs to investigate the TFs function of alleviation of salt stress by SA in pepper. There were 66 TFs belonging to 25 TF families were among the DEGs. The TF families with the largest number and the highest proposition of DEGs were MYB (11, 17%), with AP2(9, 14%), bZIP (5, 8%), HB (4, 6%), NAC (4, 6%) and Orphans (4, 6%) as following (Fig. S1). The MYB, AP2, bZIP and NAC TF families are known to play roles in plant defence responses [36]. In addition, another two TF families: C2C2-Dof and GRAS have the proposition with 5%. Furthermore, all the 11 different expressed MYBs have opposite expression patterns between NaCl vs. CK and NS vs. NaCl, and there six of them were divided into the NaCl vs. CK_down and NS vs. NaCl_up section, may five belonged to CK_up and NS vs. NaCl_down section (Table S5).

## Discussion

In previous studies, the exogenous application of proline [37], nitric oxide [38], polyamines [39] and SA [40] have been used to develop effective strategies to reduce the harmful effects of salinity on plant physiology in some species. Many studies have shown that SA functions as a growth regulator to improve salt resistance in plants. Treatments with SA have been shown to improve the nutrient status of plants, and increase the contents of some organic solutes and osmoprotectors such sugars, proline, and proteins [41]. SA involved in improving photosynthetic rate due to the K^+^ absorption [19], and capacity to increased rubisco activity [20], increasing ATP content and maintaining optimum K^+^/Na^+^ ratio in plants [21]. Nevertheless, there is little known about how much salinity or SA impact pepper growth development. Hence, we deeply investigated the impact of salt treatment (100 mM NaCl), with or/and without exogenous SA (0.15 mM) on the growth, cell morphology, osmolytes, ionic homeostasis (K^+^ and Na^+^), and primary metabolome.

### Salicylic acid improves growth state and photosynthesis under salinity

In this study, the NaCl-treated pepper leaves were yellow and shrivelled, the root growth was weakened, showing that the plant growth was inhibited under salt stress (Fig. 1). In our previous study, the biomass (including fresh wight and dry weight) of pepper seedlings were significantly weaken under salt stress treatment, and then enhanced by salt plus SA treatment [42]. One reason for the weaken growth may be the inhibition of cell division and cell elongation caused by NaCl stress (Fig. 2). In previous studies, NaCl was shown to directly affect chloroplast formation and development in cucumber, while an SA treatment increased the volume of leaf cells and resulted in a neater and denser arrangement of palisade cells, leading to more chloroplasts per unit area [43]. Therefore, leaf thickness and chloroplast and leaf microstructure can be used to indicate the tolerance of plants to salt stress and the ability of SA to relieve salt stress. Other possible reasons include reduced absorption of mineral nutrients, inhibited cytoplasmic enzyme activity, and hormone imbalances [44].

In many studies, as another important aspect of plant growth, photosynthetic capacity is reduced under salt stress often attributed to inhibition of photosynthetic rate [45]. Hence, exogenous SA application may enhance photosynthesis, which is a major controlling factor for plant growth and yield [46]. And we have previously found that the reduced net photosynthetic rate (Pn), as well as chlorophyll content under salt stress was considerable enhanced by exogenously applied SA [42], which was in agreement with some earlier studies that found that exogenous application of SA increased the photosynthetic rate in different crops, such as, thyme [47], radish [48], and maize [49]. In addition, stomatal conductance (Gs), and the ratio of internal and external CO_2_ concentration (Ci) were also dramatically affected by salinity and returned to a certain extent by SA treatment in pepper [42]. Reduction in photosynthesis under salt stress also result from chlorophyll fluorescence alteration, photosystem II photoinhibition, conformational changes in membrane-bound ATPase enzyme complex, and the reduced concentration and activity of Rubisco enzyme [50]. In addition, the most DEGs detected were enriched by GO terms in the ‘photosystem II’, ‘photosystem’, ‘Photosynthetic membrane’, ‘thylakoid’ and ‘thylakoid part’, which are all involved in photosynthesis (Fig. 6). In KEGG pathway enrichment, the most DEGs were enriched in ‘Photosynthesis-antenna proteins’, ‘Photosynthesis’, ‘Galactose metabolism’ and ‘Starch and sucrose metabolism’, which are also involved in photosynthesis (Fig. 7).

### Proline and soluble sugar accumulation responses to salinity and salicylic acid

In our study, the lipid peroxidation of pepper leaves under salt stress were reduced after SA treatment, and the proline and sugar contents were also increased (Fig. 3). Other studies showed that salt-tolerant plant varieties accumulate more proline than that of susceptible varieties, suggesting that proline plays an important role in stress tolerance [51]. The content of MDA, as an indicator of lipid peroxidation, can reflect the strength of the response to adverse conditions. Under salt stress, the contents of organic osmotic regulators such as soluble sugars increase significantly. In soybean, treatment with SA was shown to reduce salt stress and increase the contents of soluble sugars and protein organic matter permeate [44]. The application of exogenous SA can also decrease the content of malondialdehyde and enhance the ability of salt tolerance.

### ***Salicylic acid alters Na***^+^***and K***^+^***accumulation in leaves of salt stressed plants***

Na^+^ and K^+^ are the two main ions that play important role in cell homeostasis, and cells generally maintain high concentrations of K^+^ and low concentrations of Na^+^ [52, 53]. Homeostasis of Na^+^ and K^+^ is even more important under salinity. Intracellular K^+^ and Na^+^ homeostasis has an important hold on enzymes activity, maintenance of membrane potential and osmotic potential of the cell for cell volume regulation and cell function [52, 53]. Plants survival under saline condition mainly depends on the ion homeostasis, through Na^+^ moving into plant cells and reaches toxic levels it disrupts enzymes and cell function [54].

In faba bean, SA treatments were found to relieve salt stress, which were attributed to increased growth and enzyme activity, reduced Na^+^ accumulation, enhanced K^+^ absorption, and balance the Na^+^/K^+^ ratio ionic homeostasis [55]. In this study, also a higher accumulation of Na^+^, a higher Na^+^/K^+^ ratio, and decreased uptake of K^+^ were detected in pepper under salt stress treatment, while supplementation with SA alongside NaCl decreased Na^+^ accumulation and the Na^+^/K^+^ ratio, and enhanced the uptake of K^+^ (Fig. 4). Despite physicochemical similarities between Na^+^ and K^+^, the K^+^ transporters in the plasm membrane and tonoplast prefer to absorb K^+^ into cell [56]. Thus, the ability of plants to prevent salinity pressure dependent on their K^+^ acquisition may constitute an important mechanism to homeostasis maintenance, and salinity tolerance in plants, certainly in pepper. Minerals uptake is one of the main adaptations to stress tolerance, and increased mineral uptake such as K^+^ may be result in SA-induced ion transporter activity. In previous studies, overexpression of ion transporters in transgenic *Solanum lycopersicum* [57] and Switchgrass [58] led to reduced contents of toxic Na^+^ and the maintenance of ion and cell homeostasis in the transgenic plants, thereby conferring higher salt tolerance.

### *Salicylic acid relieves salt stress *via* regulation of TFs*

In general, TFs control the expression of genes during plant development and responses to stress. Here, in total 66 TFs including MYB, AP2, NAC, and bZIP were identified among the DEGs in pepper (Fig. S1). And the TFs among the DEGs with opposite expression pattern under salt without and with SA treatment, may be the key TFs involved in alleviation of salt stress by SA in pepper. In particular, the 317 DEGs (NaCl vs. CK_up and NS vs. NaCl_down) were significantly enriched in ‘sequence-specific DNA binding’, ‘transcription regulator activity’ and ‘DNA binding transcription factor activity’ by GO terms (Fig. S2). Furthermore, we identified 42 TFs among these DEGs, as the predominance in all of the DEGs (Table S3). These results indicate that the different expressed TFs under salt stress treatment and down-regulated by salt with SA treatment play an essential role in alleviation of salt stress by SA in pepper.

Among these 42 TFs, there are five MYB TFs with proportion of 12%, as well as bZIP, were identified as the regulator involved in alleviation of salt stress by salicylic acid in pepper (Fig. S3). MYB is a super family of transcription factor, which play essential roles in plant growth and development, primary and secondary metabolism, stress response and so on [59]. Over-expression of *VvMYB62* in *Arabidopsis* was shown to increase *AtNHX* expression, and increase the germination rate and salt tolerance of transgenic plants [60]. In our study, results showed that these MYB genes: *Capana04g000509*, *Capana03g000766*, *Capana12g002172*, *Capana10g001433* and *Capana06g002789* were induced to salt stress and inhibited by salt with SA, as well as five bZIP TF genes (*Capana00g005021*, *Capana01g004107*, *Capana08g001015*, *Capana04g001165* and *Capana08g002567*). MYB may play a role in SA-mediated salt response resulting in MYB expressed alterably under salt stress with SA [61, 62], and bZIP TFs such as ABI5 and ABFs (ABA-responsive element-binding factors) are involved in salt resistance by ABA signalling through phosphorylated by the SnRK2s at the plasma membrane [63]. Furthermore, four *HB* (also called HALZ, Homeobox associated leucine zipper) genes were identified among these different expressed TF genes. BBX TFs are well-known to be involved in plant development, especially light, circadian signalling and flowering in plant [64]. And our results showed that there are four *BBX* genes also found to be in these 44 TFs, and all these four *BBX* genes were induced expressed under salt stress condition, down regulated by salt with SA treatment. This indicated that BBX TFs are take part in alleviation of salt stress by SA. AP2-EREBP TFs are a kind of plant-specific TFs which have been identified 175 members in pepper [65]. And this gene family can be divided into an EREBP and AP2 subfamily based on containing one domain or two AP2-EREBP structural domains. The EREBP subfamily TFs involved in plant stress response, containing ethylene responsive element binding factors (ERF), dehydration responsive element binding (DREB), and other elements [66, 67]. Here, four AP2/ERF TFs respond to salt stress and down-regulated by salt with SA treatment (Table S5). In addition, some other TFs (Dof, GRAS, TCP, WRKY, etc.) genes were found among these DEGs of alleviation of salt stress by SA, which were detected involved in stress response and plant defence in previous studies.

## Conclusion

Salt stress causes diverse damage on pepper plant, leading to production and value loss. Several previous studies reported that the alleviation of salt stress by SA in several plants physiologically. However, the molecular mechanism of the alleviation of salt stress by SA are still rarely understood. We have integrated morphological, physiological, cytological, and transcriptomic data to explore the role of SA mitigating salt stress in pepper. We identified a number of DEGs and annotated them using the GO and KEGG databases. The results show that important DEGs which expressed induced expressed under salt stress condition, down regulated by salt with SA treatment, are enriched in ‘sequence-specific DNA binding’, ‘transcription regulator activity’ and ‘DNA binding transcription factor activity’ by GO terms. This indicated that TFs probably make a great contribution in the alleviation of salt stress by SA. Taken together, these data shed light on the molecular mechanism of TFs in plants, and provide important clues for further research on genes and networks that function to confer salt tolerance in pepper. We can also conclude that transcriptomics-assisted crop trait studies are a useful tool for studying the regulatory mechanisms of stress tolerance.

## Supplementary Information

Below is the link to the electronic supplementary material.

Supplementary file1 (Transcription factor families distribution of 901 identified DEGs.)

Supplementary file2 (GO terms Functional classification of 317 DEGs belonged to NaCl vs. CK_up and NS vs. NaCl_down section into three categories: molecular function (MF), biological process (BP), and cellular component (CC). X-axis indicates GO term. Y-axis represents the level of significance of GO term enrichment.)

Supplementary file3 (Transcription factor families’ distribution of identified DEGs belonged to NaCl vs. CK_up and NS vs. NaCl_down section.)

Supplementary file4 (XLSX 2422 KB)

Supplementary file5 (DOCX 14 KB)

## Data Availability

All data generated or analyzed during this study are included in this article (and its supplementary information files) or are available from the corresponding author on reasonable request. The genes of pepper with gene ID (like Capana12g002172) can be downloaded from the Pepper Genome Platform (PGP: http://peppergenome.snu.ac.kr/download.php).

## References

[1] MunnsGilliham RM (2015). Salinity tolerance of crops—what is the cost?. New Phytol.

[2] Boyer JS (1982). Plant productivity and environment. Science.

[3] Pan YQ, Guo H, Wang SM, Zhao B, Zhang JL, Ma Q (2016). The photosynthesis, Na(+)/K(+) homeostasis and osmotic adjustment of *Atriplex canescens* in response to salinity. Front Plant Sci.

[4] Munns R, James RA, Gilliham M, Flowers TJ, Colmer TDJFPB (2016). Tissue tolerance: an essential but elusive trait for salt-tolerant crops. Funct Plant Biol.

[5] Shabala S, Pottosin I (2014). Regulation of potassium transport in plants under hostile conditions: implications for abiotic and biotic stress tolerance. Physiol Plant.

[6] Mamrutha HM, Singh R, Sharma D, Venkatesh K, Sharma I, Kumar A, Sehgal D, Raina SN, Rajpal VR (2019). Physiological and molecular basis of abiotic stress tolerance in wheat. Genetic enhancement of crops for tolerance to abiotic stress mechanisms and approaches.

[7] Rajendran K, Tester M, Roy SJ (2009). Quantifying the three main components of salinity tolerance in cereals. Plant Cell Environ.

[8] Prusty MR, Sung-Ryul K, Ricky V, Frederickson E, James E, Diaz MGQ (2018). Newly identified wild rice accessions conferring high salt tolerance might use a tissue tolerance mechanism in leaf. Front Plant Sci.

[9] Sakhabutdinova AR, Fatkhutdinova DR, BezrukovaShakirova MVFM (2003). Salicylic acid prevents the damaging action of stress factors on wheat plants. Bulg J Plant Physiol.

[10] Wang M, Zheng Q, Shen Q, Guo S (2013). The critical role of potassium in plant stress response. Int J Mol Sci.

[11] Kang HM, Saltveit ME (2002). Chilling tolerance of maize, cucumber and rice seedling leaves and roots are differentially affected by salicylic acid. Physiol Plant.

[12] Jini D, Joseph B (2017). Physiological mechanism of salicylic acid for alleviation of salt stress in rice. Rice Sci.

[13] Miura K, Tada Y (2014). Regulation of water, salinity, and cold stress responses by salicylic acid. Front Plant Sci.

[14] Sah SK, Reddy KR, Li J (2016). Abscisic acid and abiotic stress tolerance in crop plants. Front Plant Sci.

[15] Wang LJ, Fan L, Loescher W, Duan W, Liu GJ, Cheng JS (2010). Salicylic acid alleviates decreases in photosynthesis under heat stress and accelerates recovery in grapevine leaves. BMC Plant Biol.

[16] Gunes A, Inal A, Alpaslan M, Cicek N, Guneri E, Eraslan F (2005). Effects of exogenously applied salicylic acid on the induction of multiple stress tolerance and mineral nutrition in maize (*Zea mays* L.). Archives of Agronomy and Soil Science.

[17] Khan N, Syeed S, Masood A, Nazar R, Iqbal N (2010). Application of salicylic acid increases contents of nutrients and antioxidative metabolism in mungbean and alleviates adverse effects of salinity stress. Int J Plant Biol.

[18] Misra N, Saxena P (2009). Effect of salicylic acid on proline metabolism in lentil grown under salinity stress. Plant Sci.

[19] Fayez KA, Bazaid SA (2014). Improving drought and salinity tolerance in barley by application of salicylic acid and potassium nitrate. J Saudi Soc Agric Sci.

[20] Lee SY, Damodaran PN, Roh KS (2014). Influence of salicylic acid on rubisco and rubisco activase in tobacco plant grown under sodium chloride in vitro. Saudi J Biol Sci.

[21] Hayat Q, Hayat S, Irfan M, Ahmad A (2010). Effect of exogenous salicylic acid under changing environment: a review. Environ Exp Bot.

[22] Azuma R, Ito N, Nakayama N, Suwa R, Nguyen NT, Larrinaga-Mayoral JA (2010). Fruits are more sensitive to salinity than leaves and stems in pepper plants (*Capsicum annuum* L.). Sci Hortic.

[23] Del Amor FM, Cuadra-Crespo PJFPB (2012). Plant growth-promoting bacteria as a tool to improve salinity tolerance in sweet pepper. Funct Plant Biol.

[24] Hu S, Shen Y, Chen X, Gan Y, Wang X (2013). Effects of saline water drip irrigation on soil salinity and cotton growth in an Oasis Field. Ecohydrology.

[25] Levy, Y., Lifshitz, J., Malach, Y. D., & David, Y. (1999). The Response of Several Citrus Genotypes to High-salinity Irrigation Water. *Hortscience* (pp. 878–881). A Publication of the American Society for Horticultural Science.

[26] Aktas H, Abak K, Eker S (2012). Anti-oxidative responses of salt-tolerant and salt-sensitive pepper (*Capsicum annuum* L.) genotypes grown under salt stress. J Pomol Hortic Sci.

[27] Yuan P, Zhenhua W, Lu Y (2012). Differences in cell wall components and allocation of boron to cell walls confer variations in sensitivities of *Brassica napus* cultivars to boron deficiency. Plant Soil.

[28] Lichtenthaler HK, Wellburn AR (1985). Determination of total carotenoids and chlorophylls a and b of leaf in different solvents. Biochem Soc Trans.

[29] Madhava Rao KV, Sresty TV (2000). Antioxidative parameters in the seedlings of pigeonpea (*Cajanus cajan* (L.) Millspaugh) in response to Zn and Ni stresses. Plant Sci.

[30] Bates LS, Waldren RP, Teare ID (1973). Rapid Determination of Free Proline for Water-Stress Studies. Plant Soil.

[31] El-Samad HMA, Shaddad MAK (1997). Salt tolerance of soybean cultivars. Biol Plant.

[32] Wang CM, Zhang JL, Liu XS, Li Z, Wu GQ, Cai JY (2009). *Puccinellia*
*tenuiflora* maintains a low Na^+^ level under salinity by limiting unidirectional Na^+^ influx resulting in a high selectivity for K^+^ over Na^+^. Plant Cell Environ.

[33] Audic S, Claverie JM (1997). The significance of digital gene expression profiles. Genome Res.

[34] Yang L, Smyth GK, Wei S (2014). featureCounts: an efficient general purpose program for assigning sequence reads to genomic features. Bioinformatics.

[35] Chen C, Chen H, Zhang Y, Thomas HR, Frank MH, He Y (2020). TBtools: an integrative toolkit developed for interactive analyses of big biological data. Mol Plant.

[36] Zhao S, Zhang Q, Liu M, Zhou H, Ma C, Wang P (2021). Regulation of plant responses to salt stress. Int J Mol Sci.

[37] de Freitas PAF, de Carvalho HH, Costa JH, Miranda RS, Saraiva K, de Oliveira FDB (2019). Salt acclimation in sorghum plants by exogenous proline: physiological and biochemical changes and regulation of proline metabolism. Plant Cell Rep.

[38] Kaya C, Akram NA, Ashraf M (2019). Influence of exogenously applied nitric oxide on strawberry (Fragaria x ananassa) plants grown under iron deficiency and/or saline stress. Physiol Plant.

[39] Baniasadi F, Saffari VR, Moud AM (2018). Physiological and growth responses of *Calendula officinalis* L. plants to the interaction effects of polyamines and salt stress. Sci Hortic.

[40] Youssef SM, Elhady SA, Aref RM, Riad GS (2018). Salicylic acid attenuates the adverse effects of salinity on growth and yield and enhances peroxidase isozymes expression more competently than proline and glycine betaine in cucumber plants. Gesunde Pflanzen.

[41] Wasti S, Mimouni H, Smiti S, Zid E, Ben Ahmed H (2012). Enhanced salt tolerance of tomatoes by exogenous salicylic acid applied through rooting medium. OMICS-A J Integr Biol.

[42] Wang Y, Wang L, Ma J, Lin D, Yang YJ (2020). Effects of salicylic acid on seed Germination and seedling growth of pepper under salt stress (in Chinese). North Hortic.

[43] Yildirim E, Turan M, Guvenc I (2008). Effect of foliar salicylic acid applications on growth, chlorophyll, and mineral content of cucumber grown under salt stress. J Plant Nutr.

[44] Farhangi-Abriz S, Ghassemi-Golezani KJE (2018). How can salicylic acid and jasmonic acid mitigate salt toxicity in soybean plants?. Ecotoxicol Environ Saf.

[45] Li T, Hu Y, Du X, Tang H, Shen C, Wu J (2014). Salicylic acid alleviates the adverse effects of salt stress in *Torreya grandis* cv. *Merrillii* seedlings by activating photosynthesis and enhancing antioxidant systems. PLoS ONE.

[46] Natr L, Lawlor DW (2015). Handbook of Photosynthesis.

[47] Najafian S, Khoshkhui M, Tavallali V, Saharkhiz MJ (2009). Effect of salicylic acid and salinity in thyme (*Thymus Vulgaris L.*): investigation onchanges in Gas exchange, water relations, and membrane stabilization andbiomass accumulation. Austr J Basic and Appl Sci.

[48] Nazir N, Ashraf M, Ejaz R (2001). Genomic relationships in oilseed *Brassica* with respect to salt tolerance photosynthetic capacity and ion relations. Pak J Bot.

[49] Khodary S (2003). Effect of salicylic acid on the growth, photosynthesis and carbohydrate metabolism in salt stressed maize plants. Int J Agric.

[50] Lawlor DW, Cornic G (2002). Photosynthetic carbon assimilation and associated metabolism in relation to water deficits in higher plants. Plant, Cell Environ.

[51] Kusaba S, Kano-Murakami Y, Matsuoka M, Tamaoki M, Sakamoto T, Yamaguchi I (1998). Alteration of hormone levels in transgenic tobacco plants overexpressing the rice homeobox gene OSH1. Plant Physiol.

[52] Hajiboland R, Ahmad P (2012). Effect of micronutrient deficiencies on plants stress responses. Abiotic stress responses in plants: metabolism, productivity and sustainability.

[53] Nieves-Cordones M, Alemán F, Fon M, Martínez V, Rubio F, Prasad MNV, Ahmad P (2012). K^+^ nutrition uptake and its role in environmental stress in plants. Environmental adaptations and stress tolerance of plants in the era of climate change.

[54] Ahmad P, Prasad M (2012). Environmental adaptations and stress tolerance in plants in the era of climate change.

[55] Ahmad P, Alyemeni MN, Ahanger MA, Egamberdieva D, Wijaya L, Alam P (2018). Salicylic acid (SA) induced alterations in growth, biochemical attributes and antioxidant enzyme activity in Faba Bean (*Vicia faba L.*) seedlings under NaCl toxicity. Russ J Plant Physiol.

[56] Rubio F, Nieves-Cordones M, Horie T, Shabala S (2020). Doing ‘business as usual’ comes with a cost: evaluating energy cost of maintaining plant intracellular K<sup>+</sup> homeostasis under saline conditions. New Phytol.

[57] Zhang HX, Blumwald E (2001). Transgenic salt-tolerant tomato plants accumulate salt in foliage but not in fruit. Nat Biotechnol.

[58] Huang Y, Guan C, Liu Y, Chen B, Yuan S, Cui X (2017). Enhanced growth performance and salinity tolerance in transgenic switchgrass via overexpressing vacuolar Na(+) (K(+))/H(+) antiporter Gene (PvNHX1). Front Plant Sci.

[59] Ambawat S, Sharma P, Yadav NR, Yadav RC (2013). MYB transcription factor genes as regulators for plant responses: an overview. Physiol Mol Biol Plants.

[60] Wang C (2014) Screening of salt-induced R2R3-MYB transcription factors on grapes and identification of salt resistance of VvMYB62. Shandong Agricultural University

[61] Yu Z, Duan X, Luo L, Dai S, Xia G (2020). How plant hormones mediate salt stress responses. Trends Plant Sci.

[62] Zheng J, Ma X, Zhang X (2018). Salicylic acid promotes plant growth and salt-related gene expression inL. (*Caryophyllaceae*) grown under different salt stress conditions. Physiol Mol Biol Plants.

[63] Furihata T, Maruyama K, Fujita Y, Umezawa T, Yoshida R, Shinozaki K (2006). Abscisic acid-dependent multisite phosphorylation regulates the activity of a transcription activator AREB1. Proc Natl Acad Sci U S A.

[64] Gangappa SN, Botto JF (2014). The BBX family of plant transcription factors. Trends Plant Sci.

[65] Jin JH, Wang M, Zhang HX, Khan A, Wei AM, Luo DX (2018). Genome-wide identification of the AP2/ERF transcription factor family in pepper (*Capsicum annuum* L.). Genome.

[66] Toshitsugu Nakano KS, Fujimura T, Shinshi H (2006). Genome-wide analysis of the ERF gene family in *Arabidopsis* and rice. Plant Physiol.

[67] Yoh S, Qiang L, Dubouzet JG, Hiroshi A, Kazuo S, Kazuko YS (2002). DNA-binding specificity of the ERF/AP2 domain of Arabidopsis DREBs, transcription factors involved in dehydration- and cold-inducible gene expression. Biochem Biophys Res Commun.

