# Supplementary figures and images for: Transcriptomic analysis reveals the mechanism of the alleviation of salt stress by salicylic acid in pepper (Capsicum annuum L.)
Source: Mol Biol Rep. 2022 Nov 23;50(4):3593–606. doi: 10.1007/s11033-022-08064-y (PMC10042771; doi:10.1007/s11033-022-08064-y)

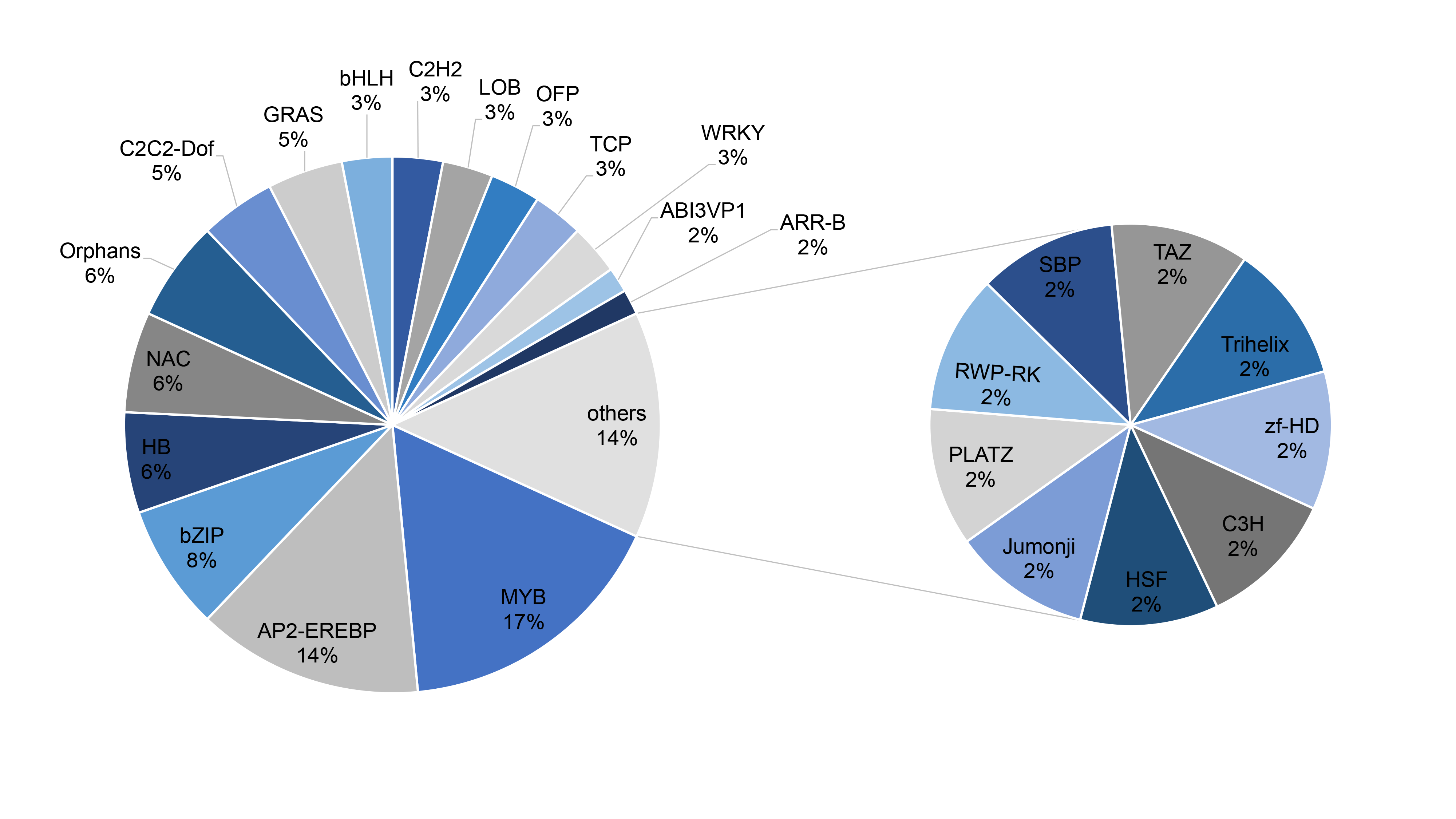

Supplement: Supplementary file 1 — Supplementary file1 (Transcription factor families distribution of 901 identified DEGs.) [file 11033_2022_8064_MOESM1_ESM.tif]

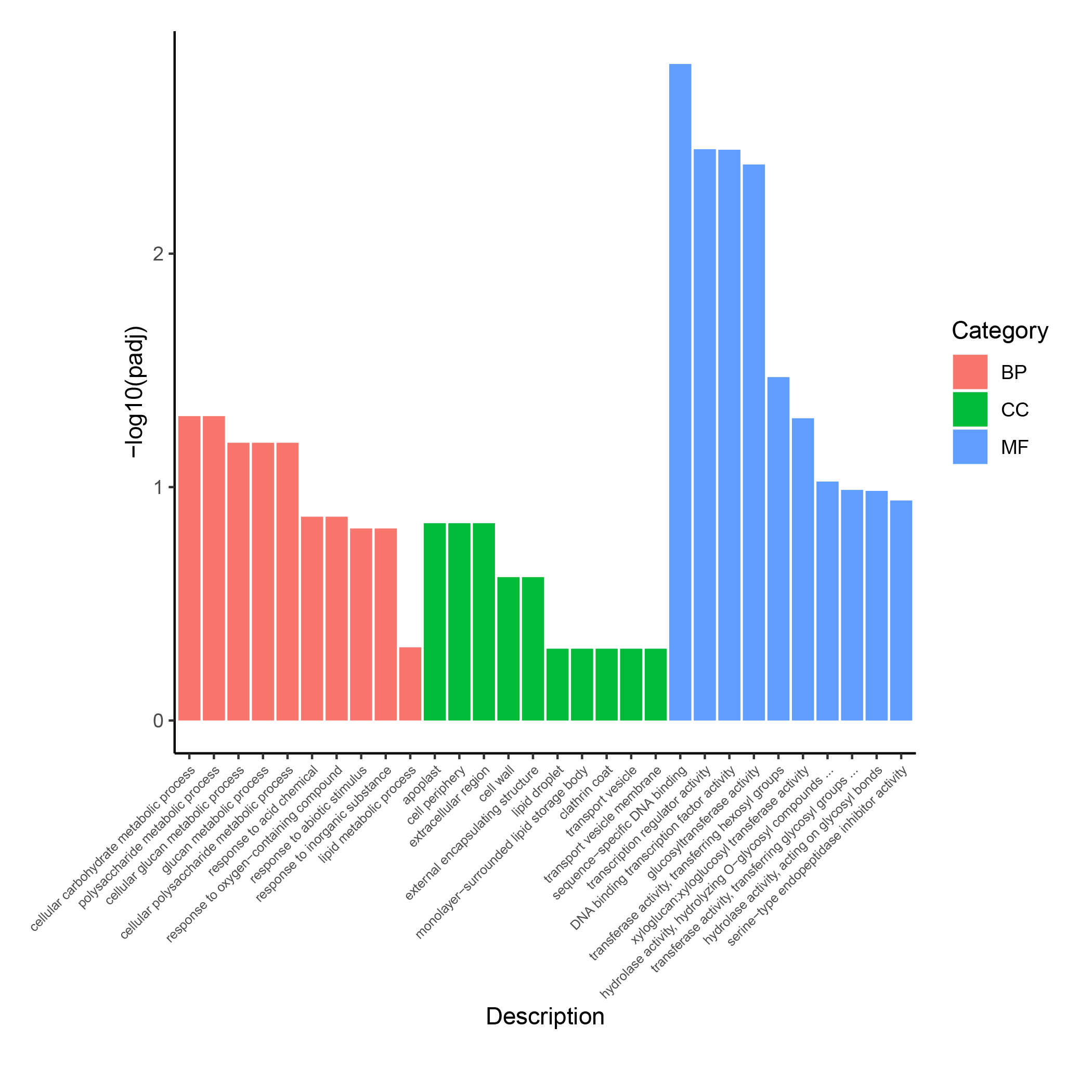

Supplement: Supplementary file 2 — Supplementary file2 (GO terms Functional classification of 317 DEGs belonged to NaCl vs. CK_up and NS vs. NaCl_down section into three categories: molecular function (MF), biological process (BP), and cellular component (CC). X-axis indicates GO term. Y-axis represents the level of significance of GO term enrichment.) [file 11033_2022_8064_MOESM2_ESM.tif]

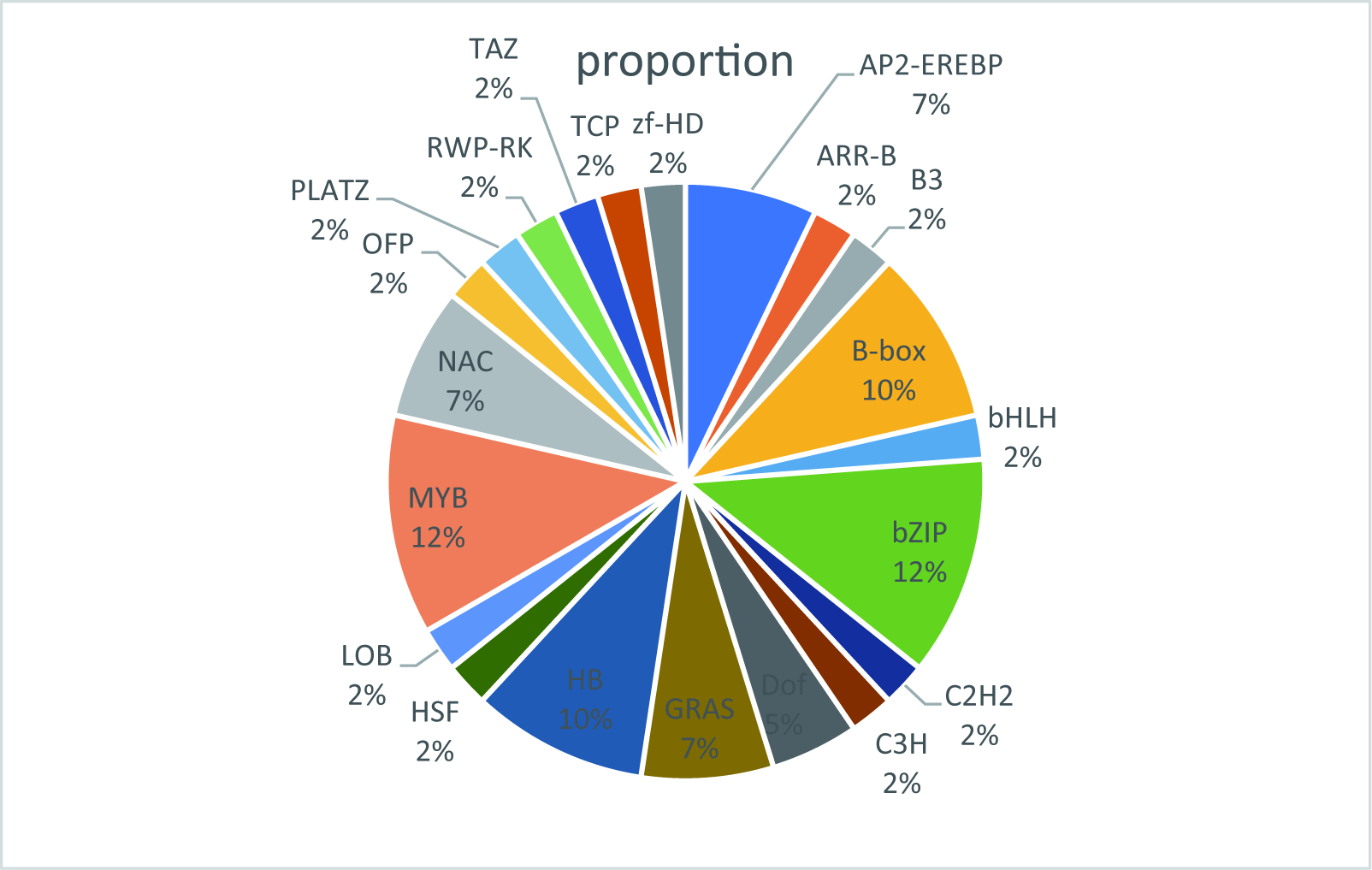

Supplement: Supplementary file 3 — Supplementary file3 (Transcription factor families’ distribution of identified DEGs belonged to NaCl vs. CK_up and NS vs. NaCl_down section.) [file 11033_2022_8064_MOESM3_ESM.tif]
