# Supplementary material for: Transcriptomic analysis reveals the mechanism of the alleviation of salt stress by salicylic acid in pepper (Capsicum annuum L.)
Source: Mol Biol Rep. 2022 Nov 23;50(4):3593–606. doi: 10.1007/s11033-022-08064-y (PMC10042771; doi:10.1007/s11033-022-08064-y)
Supplement: Supplementary file 5 — Supplementary file5 (DOCX 14 KB) [file 11033_2022_8064_MOESM5_ESM.docx]

Table S1: Summary statistics of Illumina transcriptome sequencing.

Table S2: Mapping results statistics.

Table S3: The gene information of GO enrichment.

Table S4: The gene information of KEGG enrichment.

Table S5: The information of TFs among 901 DEGs. Note: the genes marked with blue are belonged to NaCl vs. CK_up and NS vs. NaCl_down section, with yellow are belonged to NaCl vs. CK_up and NS vs. NaCl_up section, with orange are belonged to NaCl vs. CK_down and NS vs. NaCl_down section, and with green are belonged to NaCl vs. CK_down and NS vs. NaCl_up section.
